# CD47 Potentiates Inflammatory Response in Systemic Lupus Erythematosus

**DOI:** 10.3390/cells10051151

**Published:** 2021-05-10

**Authors:** Jin Kyun Park, Ye Ji Lee, Ji Soo Park, Eun Bong Lee, Yeong Wook Song

**Affiliations:** 1 Department of Molecular Medicine and Biopharmaceutical Sciences, Graduate School of Convergence Science and Technology and College of Medicine, Medical Research Center, Seoul National University, Seoul 03080, Korea; jinkyunpark@snu.ac.kr (J.K.P.); vjisuev@gmail.com (J.S.P.); leb7616@snu.ac.kr (E.B.L.); 2 Division of Rheumatology, Department of Internal Medicine, Seoul National University College of Medicine, Seoul 03080, Korea; lyj901120@naver.com

**Keywords:** systemic lupus erythematosus, CD47, SIRP-alpha, inflammatory response

## Abstract

Background: To investigate the role of CD47 in inflammatory responses in systemic lupus erythematosus (SLE). Methods: Expression of CD47 and signal regulatory protein alpha (SIRPα) by peripheral blood mononuclear cells (PBMCs) and changes in CD47 expression after exposure to SLE serum, healthy control (HC) serum, recombinant interferon (IFN)-α, or tumor necrosis factor (TNF)-α were examined. Human monocytes and THP1 cells were incubated with lipopolysaccharide (LPS), an anti-CD47 antibody, or both. TNF-α production was examined. Sera from SLE patients and HCs were screened to detect autoantibodies specific for CD47. Results: Twenty-five SLE patients and sixteen HCs were enrolled. CD47 expression by monocytes from SLE patients was higher than those from HCs (mean fluorescence intensity ± SD: 815.9 ± 269.4 vs. 511.5 ± 199.4, respectively; *p* < 0.001). CD47 expression by monocytes correlated with SLE disease activity (Spearman’s rho = 0.467, *p* = 0.019). IFN-α but not TNF-α, increased CD47 expression. Exposing monocytes to an anti-CD47 antibody plus LPS increased TNF-α production by 21.0 ± 10.9-fold (compared with 7.3 ± 5.5-fold for LPS alone). Finally, levels of autoantibodies against CD47 were higher in SLE patients than in HCs (21.4 ± 7.1 ng/mL vs. 16.1 ± 3.1 ng/mL, respectively; *p* = 0.02). Anti-CD47 antibody levels did not correlate with disease activity (Spearman’s rho = −0.11, *p* = 0.759) or CD47 expression on CD14 monocytes (Spearman’s rho = 0.079, *p* = 0.838) in patients. Conclusions: CD47 expression by monocytes is upregulated in SLE and correlates with disease activity. CD47 contributes to augmented inflammatory responses in SLE. Targeting CD47 might be a novel treatment for SLE.

## 1. Introduction

Systemic lupus erythematosus (SLE) is a chronic multi-system autoimmune inflammatory disease mediated by abnormal activation of immune cells and autoantibody production [1,2]. Unbalanced activation of both innate and adaptive immune systems leads to uncontrolled inflammation and subsequent damage to various organs [3]. Monocytes express both stimulatory receptors (e.g., Toll-like receptors (TLRs)) and inhibitory receptors [4,5]. Signal regulatory protein alpha (SIRPα) is an important regulatory receptor [6]. SIRPα interacts with integrin-associated protein cluster of differentiation (CD)-47. Although SIRPα activation by CD47 suppresses monocyte activity, the impact of CD47 activation on monocytes in SLE has not been well elucidated [7].

CD47 is a 50 kDa membrane protein that belongs to the immunoglobulin superfamily. It is expressed ubiquitously by human cells as a “marker of self” and it transduces a so-called “do not eat me” inhibitory signal to adjacent macrophages via the SIRPα receptor [8,9]. Increased CD47 expression by cancer cells helps them evade macrophage-mediated elimination [10]. Activation of CD47 itself by SIRPα is involved in regulating important cellular functions such as apoptosis, proliferation, adhesion and migration, and cell fusion [11,12,13,14]. Since CD47 activity promotes inflammatory responses such as production of interleukin (IL)-1 and increases resistance to cellular stress and survival, downregulation of CD47 expression on immune cells such as effector T cells is required for active resolution of immune responses [15]. Indeed, CD47 deficiency ameliorates autoimmune nephritis in Fas (lpr) murine lupus model and experimental autoimmune encephalitis [16].

SLE monocytes produce more tumor necrosis factor (TNF)-α, when challenged with apoptotic bodies/debris [17,18,19]. TNF-α can, in turn, increase expression of type 1 interferon (IFN), recruit neutrophils, and activate monocytes, all of which contribute to the damaging inflammatory response in organs such as the kidney [20]. The aim of this study was to investigate whether CD47 contributes to augmented inflammatory responses in SLE patients.

## 2. Materials and Methods

### 2.1. Study Population

Twenty-five patients fulfilling the 1997 revised American College of Rheumatology classification criteria for SLE were recruited at Seoul National University Hospital [21]. Disease activity at the time of blood sampling was determined using the SLE disease activity index 2000 (SLEDAI-2K) [22]. Sixteen healthy individuals without comorbidities were included as healthy controls (HCs).

### 2.2. Cell Isolation

Peripheral blood mononuclear cells (PBMCs) were isolated from heparinized peripheral venous blood by density gradient centrifugation using Ficoll-Hypaque (GE Healthcare, Princeton, NJ, USA). Cell viability was assessed by trypan blue dye exclusion. Monocytes were isolated using CD14 Microbeads (MACS, Miltenyi Biotec, Bergisch Gladbach, Germany) in accordance with the manufacturer’s instructions. Cell purity was >90%.

### 2.3. Cell Stimulation

To examine cell surface expression of CD47 and SIRPα, healthy PBMCs (10^7^ cells/mL) were incubated at 37 °C for overnight with 100 μL serum from SLE patients or HCs, IFN-α (Peprotech, Cranbury, NJ, USA), or TNF-α (Peprotech). To measure TNF-α production, PBMCs were treated for 30 min at 37 °C with a mouse anti-human CD47 monoclonal antibody (1 μg/mL; eBioscience, San Diego, CA, USA) or with an isotype control (at 1 μg/mL; BD Biosciences, San Jose, CA, USA). After treatment with GolgiStop (BD Biosciences), cells were stimulated for 5 h with LPS (at 3 ng/mL; Escherichia coli; Sigma Aldrich, Darmstadt, Germany).

### 2.4. Flow Cytometry Analysis

After blocking Fc receptors with Fc blocker (BD Biosciences), PBMCs (10^7^ cells/mL) were incubated for 30 min at 4 °C in the dark with anti-human CD3, CD14, CD47 (BD Biosciences), CD19, and SIRPα (eBioscience). For intracellular staining of cytokines, the surface-stained cells were permeabilized and fixed using Fixation/Permeabilization kit (FoxP3/Transcription factor staining set; eBioscience) and then stained with an anti-TNF-α antibody (eBioscience).

Stained cells were analyzed using an LSR Fortessa (BD Biosciences) cytometer, and data were analyzed using FlowJo software, version 8.8 (Treestar, Ashland, OR, USA).

### 2.5. Enzyme-Linked Immunosorbent Assay (ELISA)

To measure the level of anti-CD47 antibodies in serum, an ELISA plate (Nunc MaxiSorp^®^, Thermo Fisher Scientific, Waltham, MA, USA) was coated with recombinant CD47 (Novoprotein, Summit, NJ, USA) protein, blocked with PBS containing 1% BSA (Sigma Aldrich), and then incubated with serially diluted serum from SLE patients and HCs. Standard curves were generated using a mouse anti-human CD47 antibody (eBioscience), and rabbit anti-mouse IgG (Abcam, Cambridge, UK) was used as the secondary antibody. A goat anti-human IgG antibody was used (Abcam) to detect anti-CD47 antibodies in serum. Standards were measured in triplicate and all samples were measured in duplicate.

### 2.6. Hematoxylin and Eosin Staining

Paraffin sections were deparaffinized, rehydrated, and stained with Mayer’s Hematoxylin (Cancer diagnostics, Durham, NC, USA) and Eosin-Y (Cancer diagnostics). Stained sections were dehydrated and mounted (Merck, Darmstadt, Germany).

### 2.7. Immunofluorescence

Paraffin sections were deparaffinized and rehydrated. Antigens were retrieved with citrate buffer, pH 6.0 (Dako, Agilent technologies, Glostrup, Denmark). Sections were permeabilized with 0.2% Triton x-100 (Promega, Fitchburg, WI, USA) in PBS (Biosesang, Bundang, Korea), washed with PBS, blocked for 1 h with 3% bovine serum albumin (BSA) (Sigma Aldrich), 3% donkey serum (Sigma Aldrich) in PBS with 0.05% Tween 20 (Amresco, Solon, OH, USA). Sections were washed 3 times with PBS and diluted primary antibodies were added for overnight at 4 °C. Primary antibodies were as follows: mouse anti-human CD47 (Thermo Fisher Scientific), rabbit anti-human CD68 (Cell signaling technology, Danvers, MA, USA), goat anti-human CD14 (Thermo Fisher Scientific). After washing with PBS, diluted secondary antibodies were added for 1 hour at room temperature. Secondary antibodies were as follows: donkey anti-mouse IgG Alexa Fluor 488, donkey anti-rabbit IgG Alexa Fluor 594, and donkey anti-goat IgG Alexa Fluor 647 (all from Thermo Fisher Scientific). After washing, sections were mounted with mounting solution which includes DAPI (Merck).

### 2.8. Statistical Analysis

The Mann–Whitney U test or a t-test was used to compare continuous variables as appropriate. Correlation analysis was performed using Spearman’s method. All reported *p* values were two-sided. *p* values ≤ 0.05 were considered significant. All statistical analyses were performed using GraphPad Prism 5.01 (GraphPad Software Inc., La Jolla, CA, USA).

## 3. Results

### 3.1. Patient Characteristics

The mean age (± SD) of the 25 SLE patients was 38.7 ± 12.8 years. The majority of patients were female (92.0%). The median disease duration (interquartile range (IQR)) was 4.2 (0.9–14.0) years, and the median SLEDAI-2K was 6 (2–9.5). The majority of patients were taking corticosteroids and hydroxychloroquine at the time of blood sampling. Only a few patients were taking additional immunosuppressants such as azathioprine, mycophenolate mofetil, or sulfasalazine (Table 1).

### 3.2. SLE Monocytes Show Increased Expression of CD47

**Figure 1:**
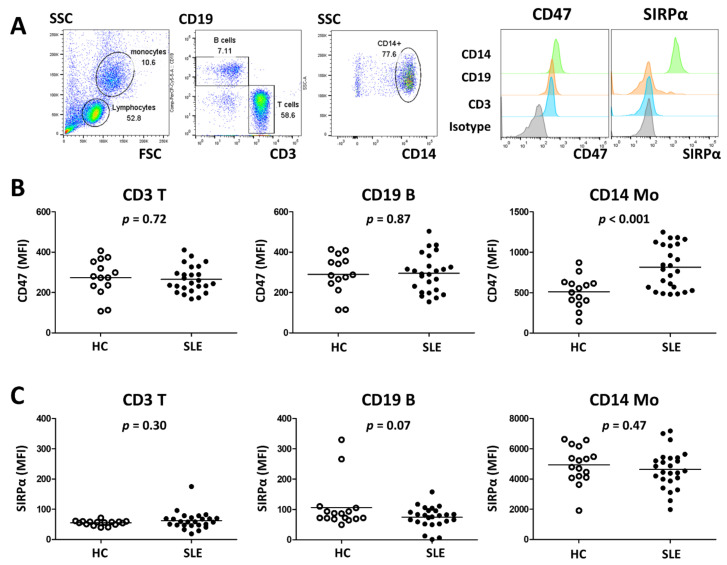
CD47 expression by SLE monocytes is upregulated. (**A**) PBMCs from 25 SLE patients and 14 healthy controls (HCs) were examined with respect to CD47 and SIRPα expression by flow cytometry. CD19+ B cells, CD3+ T cells, and CD14+ monocytes were gated (left panel) and representative surface expression of CD47 and SIRPα were depicted (right panel). CD47 expression (**B**) and SIRPα expression (**C**) on T cells, B cells and monocytes were compared between HC and SLE. *p* values were generated using t-tests. MFI, mean fluorescence intensity; Mo, monocytes.

PBMCs from SLE patients and HCs were examined for surface expression of CD47 and SIRPα. CD19+ B cells, CD3+ T cells, and CD14+ monocytes expressed CD47. CD47 expression was higher on monocytes than on B and T cells; the latter two cell populations expressed similar levels of CD47. By contrast, SIRPα was expressed mainly on CD14+ monocytes but not on B and T cells (Figure 1A).

Expression of CD47 and SIRPα on PBMCs from SLE patients (*n* = 25) and HCs (*n* = 14) was compared. CD47 expression on B and T cells did not differ between SLE patients and HCs; however, CD47 expression on SLE monocytes was higher than that on HC monocytes (mean fluorescence intensity (MFI) ± SD: 815.9 ± 269.4 vs. 511.5 ± 199.4, respectively; *p* < 0.001) (Figure 1B). SIRPα expression on B cells, T cells, and monocytes from SLE patients and HCs and were comparable (Figure 1C).

### 3.3. CD47 Expression Is Associated with SLE Disease Activity and Upregulated by Type 1 Interferon

**Figure 2:**
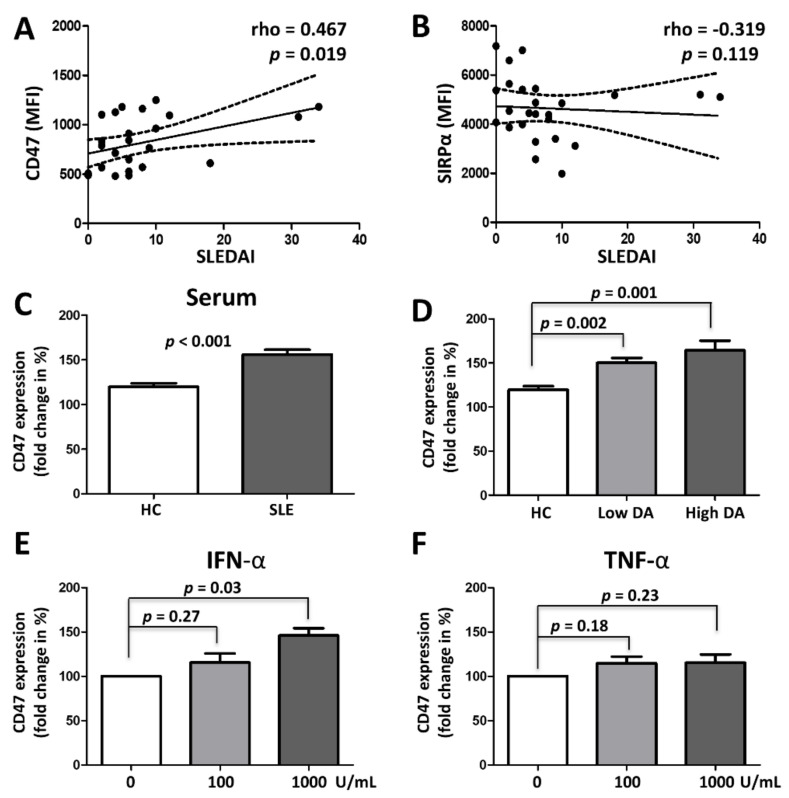
Upregulation of CD47 by SLE serum or inflammatory cytokines. Expression of CD47 (**A**) and SIRPα (**B**) on monocytes from SLE patients were correlated with their disease activity. *p* values were generated by Spearman’s correlation analysis. (**C** and **D**) Healthy PBMCs were incubated with serum from healthy controls (*n* = 6) and SLE patients (*n* = 10), and fold changes in CD47 expression on monocytes were investigated by flow cytometry analysis. (**D**) Effect of serum from patients with low (*n* = 6) and high (*n* = 4) disease activity on CD47 expression was examined. High and low disease activity were defined as SLEDAI > 12 or SLEDAI <12, respectively. (**E** and **F**) Healthy PBMCs (*n* = 3) were incubated with increasing concentrations of interferon-alpha (IFN-α) and tumor necrosis factor-alpha (TNF-α) and change in CD47 expression was examined by flow cytometry. Untreated samples served as a reference (i.e., 100%). *p* values were generated using t-tests. DA, disease activity; MFI, mean fluorescence intensity; SLE, systemic lupus erythematosus; SLEDAI, SLE disease activity index 2000.

CD47 expression on SLE monocytes correlated with SLE disease activity (SLEDAI-2K) (Spearman’s rho = 0.467, *p* = 0.019) (Figure 2A). There was no association between SIRPα expression on SLE monocytes and SLE disease activity (SLEDAI-2K) (Spearman’s rho = −0.319, *p* = 0.119) (Figure 2B).

Then, we examined whether CD47 on monocytes can be upregulated by serum from SLE or cytokines (IFN-α, TNF-α) which are known to have critical roles in SLE. Healthy PBMCs were incubated with serum from HCs (*n* = 6) and from SLE patients with low (*n* = 6) and high disease activity (*n* = 4). CD47 expression by monocytes was higher after incubation with SLE serum than with healthy serum (155.9% ± 17.5% vs. 119.8% ± 9.8%, respectively; *p* < 0.001) (Figure 2C). Subgroup analysis (according to SLE disease activity) revealed that the fold increase in CD47 expression on monocytes was higher when cells were exposed to serum from SLE patients with high disease activity (SLEDAI > 12) (mean fluorescence intensity (MFI): 164.5% ± 21.7%, *p* = 0.002) or serum from those with low disease activity (SLEDAI < 12) (MFI: 150.3% ± 13.1%, *p* = 0.001) than when cells were exposed to healthy serum (Figure 2D). IFN-α increased CD47 expression on monocytes in a dose-dependent manner (Figure 2E) whereas TNF-α did not (Figure 2F).

### 3.4. CD47 Activation Potentiates Proinflammatory Responses

**Figure 3:**
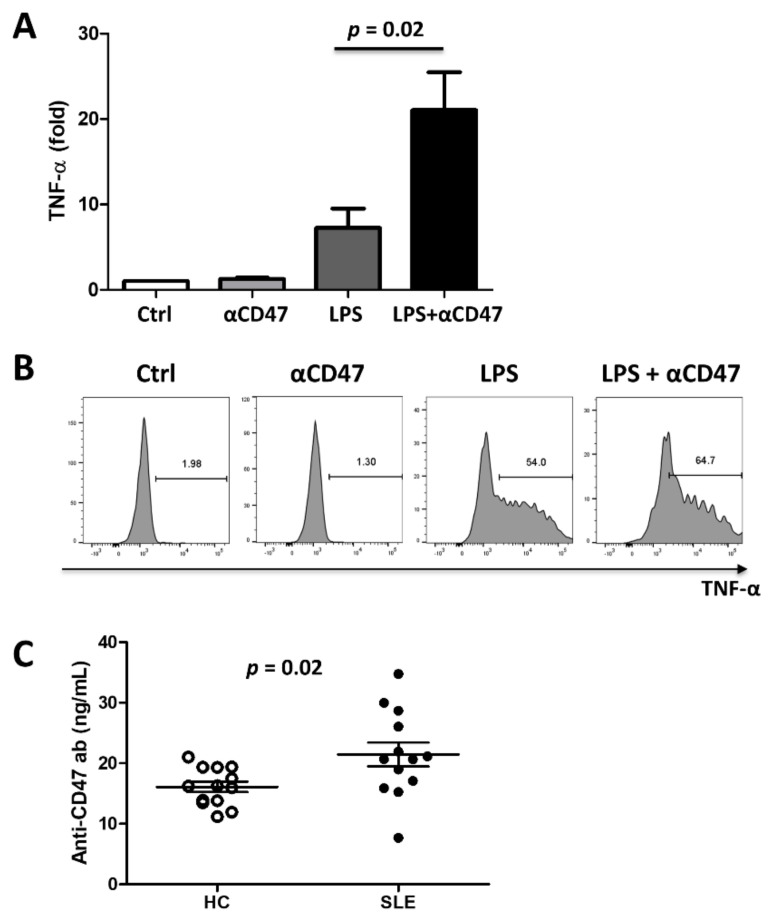
CD47 activation potentiates inflammatory responses. (**A**) PBMCs from healthy controls (*n* = 6) were stimulated for 5 hours with a mouse anti-human CD47 antibody, LPS (3 ng/mL), or both, and TNF-α production in CD14+ monocytes was measured by flow cytometry. Treatment with an anti-human CD47 antibody and LPS induced a significantly greater increase in TNF-α production in monocytes than treatment with LPS alone. (**B**) Representative flow cytometry plots are depicted. (**C**) Serum from 13 SLE patients and 13 healthy controls (HCs) was screened for anti-CD47 antibodies using ELISA. Serum anti-CD47 antibody levels were significantly higher in SLE patients than in HCs. *p* value was generated using a t-test. αCD47, anti-CD47 mouse monoclonal antibody; Ctrl, control; HC, health controls; LPS, lipopolysaccharide; SLE, systemic lupus erythematosus; TNF, tumor necrosis factor.

To investigate the role of CD47 activation during proinflammatory responses, healthy PBMCs were incubated with an anti-CD47 monoclonal antibody, LPS or both. The anti-CD47 antibody increased TNF-α production by monocytes by 1.2 ± 0.4-fold compared with the isotype control antibody (used as a reference). LPS increased TNF-α production by monocytes by 7.3 ± 5.5-fold. Interestingly, treating PBMCs with an anti-CD47 antibody and LPS together increased TNF-α production up to 21.0 ± 10.9-fold, suggesting that CD47 potentiates inflammatory responses of monocytes to LPS (Figure 3A,B).

### 3.5. Autoantibodies Directed Against CD47 Are Present in SLE

Since SLE is characterized by production of various autoantibodies, sera of SLE patients (*n* = 13) and HC (*n* = 13) were screened for the presence of autoantibodies directed against recombinant CD47 protein using an ELISA. Anti-CD47 antibody levels in serum were significantly higher in SLE patients than in HCs (21.4 ± 7.1 ng/mL vs. 16.1 ± 3.1 ng/mL, respectively; *p* = 0.02) (Figure 3C). However, anti-CD47 antibody levels in serum did not correlate with the SLEDAI (Spearman’s rho = -0.11, *p* = 0.759) or CD47 expression on CD14 monocytes (Spearman’s rho = 0.079, *p* = 0.838).

### 3.6. CD47 Expressing Macrophages Are Present in Lupus Nephritis

**Figure 4:**
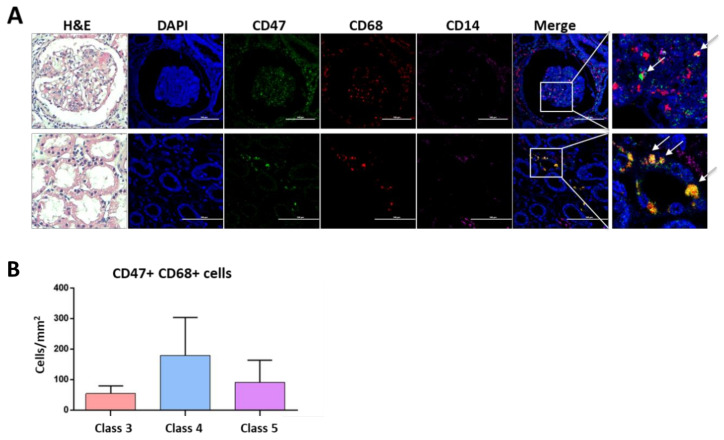
Presence of CD47 expressing macrophages in lupus nephritis. (**A**) Kidney sections from patients with lupus nephritis (LN, *n* = 7) were stained with hematoxylin and eosin (H&E) (magnification 40×). Tissue sections were stained with DAPI (blue), CD47 (AF488, green), CD14 (AF647, magenta) and CD68 (AF594, red) by immunofluorescence. Representative images are shown. Arrows indicate co-expression of CD47 and CD68. Scale bar = 100 µm. (**B**) CD47 expressing CD68 cells (number/mm^2^) in LN according to the lupus nephritis class were counted. Data are mean ± SEM.

Lastly, we investigated whether CD47 expressing cells were present in the kidney of eight patients with lupus nephritis (LN). In LN tissue, CD14+ cells were scarce as compared to CD68 positive cells. CD47 was colocalized with CD68 but not with CD14. While CD68+ cells were present in both glomerulus and tubules of the kidney, CD47 expressing CD68+ cells were mainly located in the tubules (Figure 4A). CD47+ CD68+ cells tended to be more prevalent in class 4 LN (*n* = 3) than in class 3 (*n* = 2) or class 5 of LN (*n* = 3) (Figure 4B).

## 4. Discussion

In this study, we report that CD47 expression is upregulated on monocytes from SLE patients and correlates with SLE disease activity. Activation of CD47 with an anti-CD47 antibody potentiated production of proinflammatory cytokines by monocytes in response to LPS and anti-CD47 autoantibodies were increased in sera from SLE patients. Finally, CD47+ CD68+ macrophages were present in lupus nephritis. Our findings suggest that CD47 is involved in the pathogenesis of SLE.

CD47 plays an important role in the survival of activated immune cells by providing a “do not eat me” signal to neighboring macrophages [8,9]. This may explain why activated immune cells are not immediately eliminated by adjacent activated macrophages during a proper immune response. However, prolonged survival of activated immune cells upon upregulation of CD47 might diminish or delay active resolution of inflammatory responses, leading to chronic inflammation with collateral damage [23]. Here, CD47 expression on monocytes correlates with SLE disease activity and it is upregulated by sera from SLE. A potential candidate factor in SLE serum for CD47 induction is IFN-α, a key cytokine in SLE, which is increased in the tissue and blood of patients during a lupus flare [24]. Indeed, exposure to IFN-α increased CD47 expression on monocytes, whereas TNF-α, a major cytokine produced during inflammatory responses to bacterial infections and rheumatoid arthritis, did not (Figure 2). Therefore, CD47 upregulation might not be a general feature of immune responses. Rather, it might be relatively specific to diseases, in which type 1 IFN plays a key role.

It is striking that CD47 ligation potentiated proinflammatory responses; when monocytes were treated with an anti-CD47 antibody, they produced more TNF-α in response to LPS, while CD47 activation alone did not induce any significant TNF-α production (Figure 3). Anti-CD47 antibody levels varied between patients and they did not correlate with disease activity or CD47 expression on CD14 monocytes in SLE patients. The absent correlation between levels of autoantibodies and disease activity is not unusual in diverse rheumatic diseases; levels of rheumatoid factor (RF) and autoantibodies against citrullinated proteins (ACPA) in rheumatoid arthritis, and antiphospholipid antibody titers in antiphospholipid syndrome do not correlate with disease activity, although RF/ACPA and antiphospholipid promote joint destruction and thrombus formation, respectively [25]. A two-hit hypothesis might explain why certain disease manifestations of autoantibodies occur rarely in spite of their persistent presence [26]. Here, anti-CD47 antibodies might potentiate (but not induce) inflammatory response only in the presence of a second hit such as LPS.

Several reports suggest that CD47 is crucial in the development of certain autoimmune diseases. Blockade of CD47 ameliorates encephalitis by suppressing IL-1-mediated infiltration of Th17 cells and CD47 knockout mice are refractory to development of experimental autoimmune encephalomyelitis [27,28]. In a murine lupus model, CD47 deficiency improves autoimmune nephritis by suppressing IgG autoantibody production [16]. Here, we show that CD47 expressing cells were present in the tubules of lupus nephritis and their level of the infiltrating cells tended to be higher in LN 4 (Figure 4). However, it is subject to further investigation, whether CD47 expression pattern observed in LN is specific for SLE or it is a general feature in other inflammatory kidney diseases.

**Figure 5:**
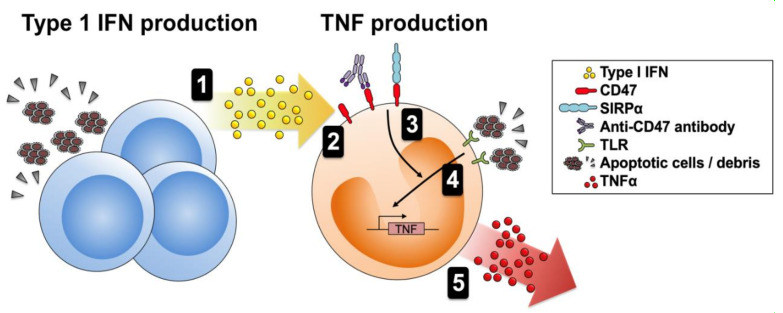
Proposed role for CD47 in SLE. (**1**) Immune and non-immune cells produce type 1 IFN when they encounter stimuli such as nucleic acids released from apoptotic debris. (**2**) Type 1 IFN increases CD47 expression by monocytes. (**3**) CD47 is activated by circulating autoantibodies; it then activates MAPK signaling components (including ERK, JNK, and p38). (**4**) LPS or other ligands bind to TLRs and activate NF-κB signaling. (**5**) Activated MAPK promotes transcription (and stability) and translation of TNF-α mRNA, leading to enhanced production of TNF-α protein. TLR, Toll-like receptor; LPS, lipopolysaccharide; TNF, tumor necrosis factor; IFN, interferon.

SLE is a prototype autoimmune disease producing multiple autoantibodies [1]. Thus, it is possible that SLE patients produce autoantibodies directed against CD47 and these anti-CD47 autoantibodies interact with CD47 in a subset of SLE patients. In this study, SLE patients showed higher levels of anti-CD47 autoantibody than HCs (Figure 3). However, there was no correlation between anti-CD47 antibody levels and SLE disease activity (data not shown). Taken together, we hypothesize that immune or non-immune cells produce a high level of type 1 IFN which upregulates CD47 expression on monocytes. Circulating anti-CD47 autoantibodies bind to CD47 on monocytes/macrophages and this triggers the activation of signaling which potentiates monocytes/macrophage response via mitogen-activated protein kinase (MAPK) (Supplementary Figure S1A–E). The pre-activated monocytes respond to a second inflammatory stimulus such as LPS with higher production of TNF-α, which contributes to tissue damaging inflammatory response (Figure 5).

In addition, anti-CD47 antibody can potentially interrupt the inhibitory “do not eat me” signaling of CD47–SIRPα interaction. While IFN activated monocytes with increased CD47 expression might still provide enough inhibitory signaling, lymphocytes such as T and B cells without CD47 upregulation might be more susceptible to removal by activated monocytes/macrophages which could lead to common lymphopenia in SLE patients. Furthermore, CD47 upregulation can inhibit excessive inflammation as a compensatory mechanism.

Anti-CD47 antibodies could have also therapeutic consequences. B cell depletion therapy with anti-CD20 antibody has been successful in a subset of SLE patients [29]. Gallagher et al. reported that blocking CD47 enhanced anti-CD19 antibody-mediated phagocytosis of both lymphoma and normal B cells, suggesting that concomitant CD47 blockade may be used to potentiate B cell depletion therapy [30]. Accordingly, anti-CD47 antibody could have paradoxical effects in SLE patients; anti-CD47 antibody might promote pro-inflammatory response whereas it can enhance the treatment response to B cell depletion with anti-CD20 antibodies. A similar effect of B-cell activating factor (BAFF) was observed in rheumatoid arthritis; BAFF activates B cells and increases disease activity, but it can negatively impact humoral response in combination with methotrexate via increasing extra-cellular anti-inflammatory adenosine concentration [31]. The potential therapeutic role of anti-CD47 antibody in SLE treatment needs further investigation.

This study has several limitations. The exact mechanism by which CD47 expression by monocytes is upregulated by type 1 IFN in SLE needs further investigation. Additionally, it is not clear whether CD47-expressing monocytes contribute directly to tissue damage in vivo or whether they are innocent bystanders. The protein that links CD47 to MAPK also needs to be identified. It is important to emphasize that monocytes express both stimulatory CD47 and inhibitory SIRPα on their surface. Therefore, the sum or balance of both signals might ultimately determine the final impact of the CD47 and SIRPα on cellular responses. As an example, net suppression of the CD47–SIRPα interaction might be associated with immune deficiency, leading to increased susceptibility of SLE patients to infection and cancer [32,33,34]. All this highlights the complexity of immune regulation and importance of the fine balance between stimulatory and inhibitory signals in immune regulation.

In conclusion, CD47 expression on monocytes is upregulated from SLE patients and CD47 activation potentiates proinflammatory responses of monocytes with increased TNF-α production. Targeting CD47 might offer a novel therapeutic opportunity in patients with SLE.

## Figures and Tables

**Table 1:** Baseline characteristics of the 25 SLE patients and 16 heathy controls.

	SLE Patients (*n* = 25)	HCs (*n* = 16)
Age, years	38.7 ± 12.8	28.8 ± 4.2
Female, *n* (%)	23 (92.0)	12 (75)
SLE duration, years	4.2 (0.9–14.0)	
White blood cell, ×10^3^/μL	5.7 ± 2.9	
Hematocrit, %	38.6 (34.5–39.9)	
Platelet, ×10^3^/μL	190.7 ± 71.0	
ESR, mm/hour	19 (14.5–33.0)	
Anti-dsDNA, IU/mL (ref: 0–10 IU/mL)	16.7 (7.7–40.5)	
C3, mg/dL (ref: 83–193 mg/dL)	86.0 (57.5–98.7)	
C4, mg/dL (ref: 15–57 mg/dL)	14.5 (7.0–16.7)	
SLEDAI-2K	6 (2–9.5)	
Treatment		
Corticosteroids	20 (80.0)	
Prednisolone equivalent, mg/day	5 (0–125)	
Hydroxychloroquine	16 (64.0)	
Azathioprine	2 (8.0)	
Mycophenolate mofetil	1 (4.0)	
Sulfasalazine	1 (4.0)	
NSAIDs	6 (24.0)	

Data are expressed as the mean ± SD, median (IQR), or *n* (%). ESR, erythrocyte sedimentation rate; HC, heathy control; IQR, interquartile range; NSAIDs, nonsteroidal anti-inflammatory drugs; ref, reference range; SLE, systemic lupus erythematosus; SLEDAI-2K, SLE disease activity index 2000.

## Data Availability

The datasets generated during and/or analyzed during the current study are available from the corresponding author on reasonable request.

## Supplementary Materials

The following are available online at https://www.mdpi.com/article/10.3390/cells10051151/s1, Figure S1: CD47 activation activates MAPK signaling.
