# Supplementary material for: CD47 Potentiates Inflammatory Response in Systemic Lupus Erythematosus
Source: Cells. 2021 May 10;10(5):1151. doi: 10.3390/cells10051151 (PMC8151692; doi:10.3390/cells10051151)
Supplement: Supplementary file 1 [file cells-10-01151-s001.zip › cells-1168661-supplementary.pdf]

cells

MDPI

Supplementary Material

CD47 Potentiates Inflammatory Response in Systemic Lupus Erythematosus

Jin Kyun Park [1,2,†], Ye Ji Lee [2,†], Ji Soo Park [1], Eun Bong Lee [1,2], and Yeong Wook Song [1,2,*]

[1] Department of Molecular Medicine and Biopharmaceutical Sciences, Graduate School of Convergence Science and Technology and College of Medicine, Medical Research Center, Seoul National University, Seoul, Korea; jinkyunpark@snu.ac.kr (J.K.P.); vjisuev@gmail.com (J.S.P.); leb7616@snu.ac.kr (E.B.L.)
[2] Division of Rheumatology, Department of Internal Medicine, Seoul National University College of Medicine, Seoul, Korea; lyj901120@naver.com
* Correspondence: ysong@snu.ac.kr; Tel: + 82-2-2072-4765
† Both authors contributed equally to work

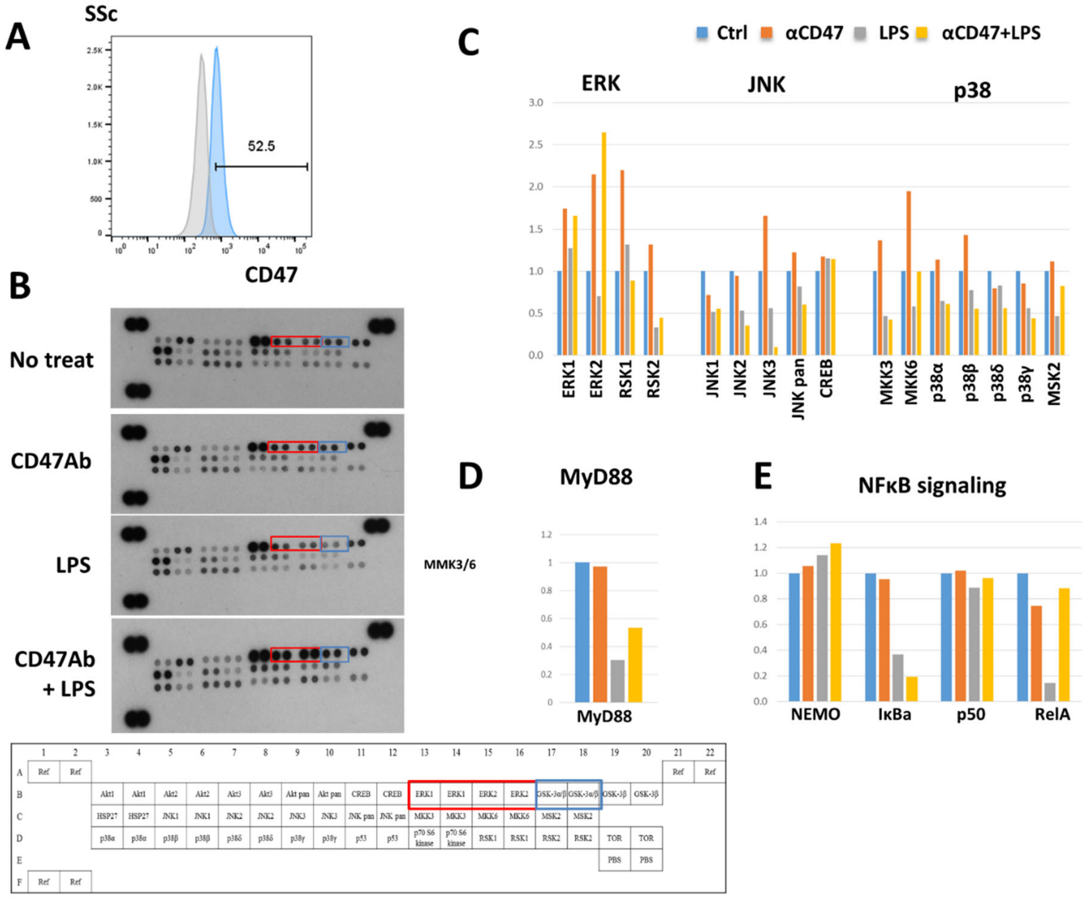

Figure S1. CD47 activation activates MAPK signaling. (**A**) Expression of CD47 on THP1 cells was examined by flow cytometry. (**B**,**C**) THP1 cells were treated with 1 µg/mL anti-CD47 antibody and/or 3 ng/mL LPS for 30 min and phospho-MAPK array was performed. (**B**) Image of phospho-MAKP-array blot with the array map (bottom panel) is shown. Phosphorylation of ERK (red rectangle) and GSK-3 (blue rectangle) is marked. (**C**) Densitometry analysis of the phospho-MAKP-array according to the 3 major arms of MAPK pathways (i.e., ERK, JNK, p38). (**D**,**E**) Densitometry analysis of the phosphor-MyD88 and phospho-NFκB array after treatment with CD47 and/or LPS is shown. Phosphorylation is shown as fold change compared to no treatment. Ctrl, control; LPS, lipopolysaccharide; SSc, Side scatter.
